# Risk of depressive symptoms before and after the first hospitalisation for cancer: Evidence from a 16-year cohort study in the Czech Republic

**DOI:** 10.1016/j.jad.2020.06.070

**Published:** 2020-11-01

**Authors:** Wentian Lu, Hynek Pikhart, Anne Peasey, Ruzena Kubinova, Alexandra Pitman, Martin Bobak

**Affiliations:** a Research Department of Epidemiology and Public Health, University College London, 1-19 Torrington Place, London, United Kingdom; b Centre for Environmental Health Monitoring, National Institute of Public Health, Prague, Czech Republic; c UCL Division of Psychiatry, University College London, London, United Kingdom; d Camden and Islington NHS Foundation Trust, London, United Kingdom

**Keywords:** Cancer, Depression, Hospitalisation, Czech republic

## Abstract

**Background:**

Whether depression risk starts increasing before cancer diagnosis, and whether cancer is an independent risk factor for depression, remain unclear. We aimed to quantify the risk of depressive symptoms before and after the first hospitalisation for cancer (as a proxy for cancer diagnosis) amongst patients with cancer.

**Methods:**

We linked cohort data with national hospitalisation records in the Czech Republic. We followed 1056 incident cancer cases for up to 15 years before and 15 years after the first hospitalisation for cancer. Depressive symptoms were measured using the Centre for Epidemiological Studies-Depression (CES-D) scale. We used multilevel ordered logistic regression to assess the relationship between follow-up years (pre- and post-hospitalisation) and depressive symptoms amongst incident cancer cases. Propensity Score Matching was employed to match each case with a cancer-free control, to test the independent effect of cancer on depressive symptoms over time.

**Results:**

Per one year of follow-up (whether pre- or post- hospitalisation) was associated with 1.07 (1.05–1.10) times more likely to have high severity of depressive symptoms amongst patients with cancer. The probability of having high severity of depressive symptoms increased from 25% at five years before hospitalisation to 33% at 7.5 years after hospitalisation. In parallel analyses amongst matched cancer-free controls, the risk of depressive symptoms had no significant changes during follow-up.

**Limitations:**

Stratified analyses based on cancer types and stages of malignancy were infeasible.

**Conclusions:**

The excess risk of depressive symptoms was apparent five years prior to the first hospitalisation for cancer. Using cancer-free matched controls, we confirmed that cancer was an independent predictor of depressive symptoms.

## Highlights

• The risk of depressive symptoms starts increasing five years prior to the first hospitalisation for cancer.
• This may be due to pre-diagnosis neuropsychiatric or biopsychosocial processes.
• This excess risk continued for up to 7.5 years after the first hospitalisation for cancer.
• Cancer is an independent risk factor for depressive symptoms.
• Early psychological assessment for patients diagnosed with cancer is needed.

## 1 Introduction

There is evidence that the prevalence of mental disorders amongst patients with cancer is higher than that amongst the general population, and that survival rates amongst patients with cancer and co-morbid anxiety or depression are significantly lower than those for the general population (Batty et al., 2017; Kisely et al., 2013; Pitman et al., 2018; Zhu et al., 2017). Clinically, depression in patients with cancer tends to be under-recognised, with depressive symptoms such as anorexia, weight loss, fatigue and insomnia often attributed to the somatic effects of cancer rather than depression (Lloyd-Williams, 2000). The World Health Organization (WHO) suggests that the risk of mental disorders in patients with cancers is routinely overlooked and should be better understood (World Health Organization, 2017).

Previous work has shown an excess risk of mental disorders (Dalton et al., 2009; Lu et al., 2016; Mallet et al., 2018; Suppli et al., 2014), and of suicide (Henson et al., 2018), after cancer diagnosis, with risks more marked in cancers with poor prognosis. For example, a population-based study in England indicated that patients with mesothelioma had the highest suicide risk amongst all patients after cancer diagnosis (Henson et al., 2018). A Danish registry-based study found a general pattern of an increased risk of depression in the first year after cancer diagnosis, with decreasing but still significant excess risk in subsequent years for most types of cancer (Dalton et al., 2009). A recent study reported that the risk of mental disorders after cancer diagnosis in the United States (US) was significantly higher amongst patients with a prior history of mental disorders compared with those without psychiatric histories (Mallet et al., 2018). A Swedish registry-based study suggested that risk of mental disorders both before and after cancer diagnosis increased to a greater extent amongst patients with cancers of poor prognosis (i.e. lung and colorectal cancers) compared with patients with other cancers (i.e. breast cancer) (Lu et al., 2016). Generally risk of depression and anxiety applies at all points in the cancer trajectory, whether in curative or palliative treatment (Burgess et al., 2005; Fallowfield et al., 1990; Mitchell et al., 2011).

The onset of depression in relation to cancer diagnosis requires careful study, as it provides clues as to the aetiology of depression in the cancer context, including the putative effect of cancer-related inflammation on mental disorders (Messay et al., 2012). Previous work has shown that the excess risk of depression is apparent in the year before cancer diagnosis, corresponding with the period of cancer diagnostic workup (Lu et al., 2016). This suggests that direct neuropsychiatric effects may be involved even before an awareness of cancer diagnosis, at which point predominant explanations for depression or anxiety involve biopsychosocial processes (Pitman et al., 2018). The majority of work describing the association between cancer and depression derives from high-income countries, including the US, England, Denmark, Australia and Sweden (Batty et al., 2017; Burgess et al., 2005; Dalton et al., 2009; Fallowfield et al., 1990; Henson et al., 2018; Kisely et al., 2013; Lu et al., 2016; Suppli et al., 2014; Zhu et al., 2017). However, the generalisability of these findings to more recent years or amongst other populations is questionable.

To address the research gap, our 16-year longitudinal study aimed to assess the risk of depressive symptoms before and after the first hospitalisation for cancer (as a proxy for cancer diagnosis) in the Czech Republic. We investigated whether and when depressive symptoms occur before the first hospitalisation for cancer. We also used matched controls to evaluate the independent effect of cancer on depressive symptoms.

## 2 Methods

### 2.1 Data

**Fig. 1:**
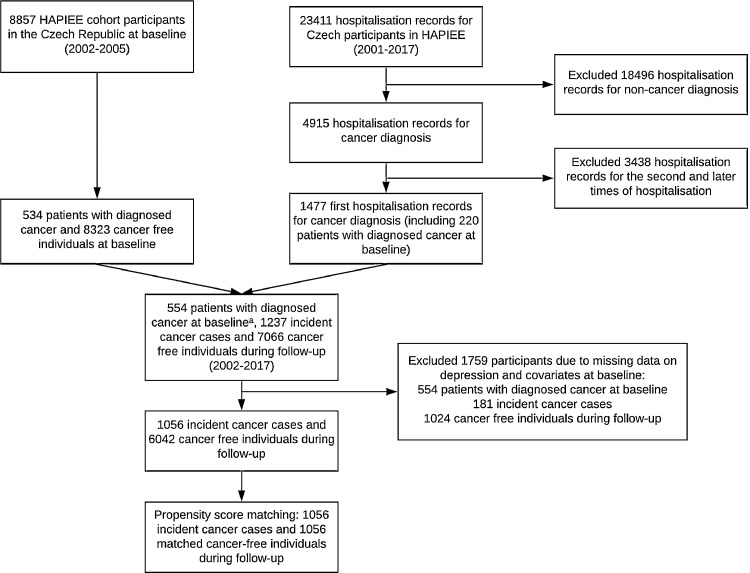
Flowchart of sample selection ^a^20 individuals with baseline cancer-free records were re-categorised as patients with cancer at baseline since they were diagnosed with cancer before entering the HAPIEE cohort according to their first hospitalisation records. HAPIEE: Health, Alcohol and Psychosocial factors In Eastern Europe.

Data were from the Health, Alcohol and Psychosocial factors In Eastern Europe (HAPIEE) study – Czech cohort, a prospective cohort study of a representative sample of 8857 individuals (response rate: 82%) aged 45–69 years at baseline in the Czech Republic (Peasey et al., 2006). Random samples stratified by gender and five-year age groups were selected from population registers. Written informed consent was obtained from all participants. The study received ethical approval from the ethics committee at University College London, UK (99/0081). We used data from baseline wave (2002–2005), wave 2 (2006–2008) and follow-up postal surveys in 2009, 2012, 2013, 2016 and 2017. The information on cancer diagnosis was obtained from the national hospitalisation records (2001–2017) of all participants, which contain information on, for example, dates of admission and discharge, ICD codes of primary, operation and other diagnosis, and whether being hospitalised for the first time for each diagnosis. We included 1056 (473 women) incident cancer cases (2003–2017) (Supplementary Figure S1), who were matched with population-based controls (matching ratio: 1:1) using Propensity Score Matching (PSM) (Rosenbaum and Rubin, 1983). Fig. 1 illustrates the detailed procedure of sample selection.

### 2.2 Variables

#### 2.2.1 Depressive symptoms

Depressive symptoms were measured using the Centre for Epidemiological Studies-Depression (CES-D) scale (Radloff, 1977), and used as a time-varying outcome. The format of the CES-D scale used at baseline (CES-D-20; original 20-item measure, 4-category response) differed at Wave 2 (2006–2008), the 2009 postal surveys (CES-D-10; 10-item measure, Boston scale; 2-category response), and the 2012–2017 postal surveys (CES-D-10; 10-item measure, Andresen scale; 4-category response) (Kohout et al., 1993; Mohebbi et al., 2018). Cronbach's alpha values were all above 0.70, indicating acceptable reliability (Heale and Twycross, 2015). To maintain the integrity of the scale, up to four, one and one missing items, respectively, were allowed for calculating the sum scores based on original, Boston and Andresen scales. We substituted the mean values for missing items then summed all items (Kohout et al., 1993). For comparability of results over time, we organised sum scores into tertiles based on the entire sample size. High severity of depressive symptoms was defined as the highest tertile of CES-D scores.

#### 2.2.2 Incident cancer cases

We identified incident cancer cases in cancer-free individuals at baseline, measured as the first ever hospitalisation for any cancer (ICD-10: C00–C97) recorded on the national hospitalisation register during follow-up (as a proxy for cancer diagnosis), on a date later than the dates of baseline interview. Supplementary Figure S1 and Figure S2 illustrate the numbers of incident cancer cases (2003–2017), and the numbers of each type of cancer, respectively.

#### 2.2.3 Follow-up years

We calculated each participant's number of years of follow-up using the date of depressive symptom measurement in each wave of data collection minus the date of the first hospitalisation for any cancer. The date of the first cancer hospitalisation (a proxy for diagnostic year) was set at zero. Negative and positive values designated pre-hospitalised (−15.0 to −0.1) and post-hospitalised (0.1 to 15.0) years. Supplementary Figure S3 shows a normal distribution of follow-up years.

#### 2.2.4 Confounders

We selected confounders based on previous evidence (Batty et al., 2017; Bortolato et al., 2017; Burgess et al., 2005; World Health Organization, 2017): baseline gender; age; marital status; education; smoking; alcohol use; fruit consumption; vegetable consumption; weekly hours of physical activity; diagnosed cardiovascular disease (CVD), diabetes and chronic respiratory diseases; and Body Mass Index.

### 2.3 Statistical methods

We conducted univariate analysis of baseline relationships between confounders and depressive symptoms. We used multilevel ordered logistic regression to assess the longitudinal relationship between follow-up years and depressive symptoms amongst incident cancer cases, allowing for random intercepts. Multilevel modelling can handle attrition and wave non-response (which allows us to include incident cancer cases who died during the post-diagnostic period), unequal time spaces, and the inclusion of time-varying and between-individual covariates that are either continuous or discrete measures (Curran et al., 2010).

A time-cohort model (repeat follow-up year model controlling for baseline age group) was estimated with full adjustment. We included year as a linear, quadratic, and a cubic term, to detect non-linear effects. We predicted the overall average probability of being in the highest tertile of CES-D scores in each year, considering both fixed and random effects based on this model. We fitted curves using kernel-weighted local polynomial smoothing to explore non-linear effects of follow-up years, allowing the data to “speak for themselves” by fitting the response to a polynomial form of the regressor via locally weighted least squares (Gasser and Müller, 1979). We produced curves within a thirty-year range (15 pre-hospitalised years and 15 post-hospitalised years).

To test the independent effect of cancer on depressive symptoms over time, we used PSM to match each incident cancer case with a cancer-free participant based on similar propensity scores obtained from a logistic regression model adjusting for all aforementioned confounders (Rosenbaum and Rubin, 1983). The nearest neighbour matching was used (calliper bound=0.04, mean bias=1.8%) (Rubin, 1973). As a result, the observed baseline characteristics became very similar between incident cancer cases and those of cancer-free controls (Supplementary Table S1). In order to compare risk of depressive symptoms before and after a hypothetical non-cancer diagnosis date in cancer-free controls, each control was allocated a non-exposure data, which was the date of the first cancer hospitalisation of his or her matched treated participant. We assessed the unadjusted relationships between follow-up years and depressive symptoms in both groups. We also predicted the overall probabilities of being in the highest tertile of CES-D scores after year=0 in both groups.

We conducted a *post hoc* analysis to confirm whether the risk of depressive symptoms did indeed increase in the five years prior to the first cancer hospitalisation. For this, we applied fully-adjusted piecewise regression with three segments separated by two “knots” (at year −5 and 0) (Ryan and Porth, 2007), to quantify the slope changes of the probability of being in the highest tertile of CES-D scores. Three independent variables were included in piecewise regression, reflecting three segments: ‘6–15 years before hospitalisation’ ‘1–4 years before hospitalisation’ and ‘1–15 years after hospitalisation’.

#### 2.3.1 Sensitivity analyses

We compared baseline characteristics between the analysed (*N* = 1056) and excluded incident cancer cases (*N* = 181), to explore whether missing data were likely to bias findings. We also employed another harmonisation strategy – converting the CES-D sum scores into z-scores (mean [S.D.] =0 [1]) and re-run the fully-adjusted model using the continuous z-scores, which helps predict the overall changes of depressive symptoms over time, and make further comparison with other samples.

All analyses were performed using Stata SE 15 (StataCorp, 2017), with a p-value threshold of <0.05 for statistical significance.

## 3 Results

### 3.1 Baseline sample characteristics

Table 1 presents baseline sample characteristics amongst incident cancer cases. Over half (55%) this sample were men. Participants were mainly aged between 50 and 69 years, married or cohabiting, and had vocational or secondary education at baseline. Around 30% of participants were current smokers. Around 15% of participants consumed alcohol more than 5 times per week. Participants consumed a mean of 3.48 (S.D. = 3.52) and 3.13 (S.D. = 2.25) portions of fruit and vegetable per day, respectively. The mean hours spent on physical activity per week were 13.49, but with wide variation (S.D. = 12.50). The majority of the participants were diagnosed with diabetes, CVD, or chronic respiratory diseases, and 45% and 34% were pre-obese and obese, respectively.

**Table 1:** Baseline sample characteristics amongst incident cancer cases (*N* = 1056).

Risk factors	Total	CES-D scores – lowest tertile (39.68%)	CES-D scores – middle tertile (33.62%)	CES-D scores – highest tertile (26.70%)
Gender (%)				
Men	55.21	43.57	33.79	22.64
Women	44.79	34.88	33.40	31.71
Age (%)				
<50 years	6.16	38.46	26.15	35.38
50–59 years	32.10	38.35	32.15	29.50
60–69 years	59.09	40.06	35.26	24.68
≥70 years	2.65	50.00	32.14	17.86
Marital status (%)				
Married/Cohabiting	74.91	43.24	34.01	22.76
Single/Divorced/Separated	14.20	30.67	35.33	34.00
Widowed	10.89	26.96	28.70	44.35
Education (%)				
University degree	13.83	50.68	26.03	23.29
Secondary education	35.04	41.62	33.51	24.86
Vocational education	38.26	37.87	36.39	25.74
Primary education or below	12.88	27.94	33.82	38.24
Smoking (%)				
Non-smokers	37.88	38.25	34.25	27.50
Previous smokers	32.10	39.82	36.87	23.30
Current smokers (<1 cigarette per day)	1.80	36.84	47.37	15.79
Current smokers (≥1 cigarette per day)	28.22	41.61	28.19	30.20
Alcohol consumption (%)				
Non-alcohol consumers	12.31	29.23	36.15	34.62
<1 time per month	25.28	40.82	31.46	27.72
1–3 times per month	20.64	38.53	33.94	27.52
1–4 times per week	26.33	40.29	33.45	26.26
≥5 times per week	15.44	46.63	34.97	18.40
Physical activity (hours per week)				
Mean (S.D.)	13.49 (12.50)	14.07 (12.54)	13.44 (12.69)	12.70 (12.21)
Fruit consumption (portions per day)				
Mean (S.D.)	3.48 (3.52)	3.59 (3.51)	3.44 (3.98)	3.37 (2.84)
Vegetable consumption (portions per day)				
Mean (S.D.)	3.13 (2.25)	3.17 (2.00)	3.08 (2.44)	3.14 (2.35)
Diabetes (%)				
No	86.84	40.13	33.48	26.39
Yes	13.16	36.69	34.53	28.78
Cardiovascular disease (%)				
No	83.62	40.77	33.41	25.82
Yes	16.38	34.10	34.68	31.21
Chronic respiratory diseases (%)				
No	83.05	42.42	33.07	24.52
Yes	16.95	26.26	36.31	37.43
Body Mass Index (%)				
Normal weight (BMI 18.5 to 24.9)	20.83	40.91	26.36	32.73
Pre-obesity (BMI 25.0 to 29.9)	44.79	39.53	36.15	24.31
Obesity (BMI ≥ 30.0)	34.38	39.12	34.71	26.17

CES-D: Centre for Epidemiological Studies-Depression; S.D.: Standard Deviation; BMI: Body Mass Index.

Our sample contained more than twenty types of cancer. The sample sizes of different types of cancer were diverse (Supplementary Figure S2). For example, there were 102, 118 and 141 incident cases of lung (C33, C34), breast (C50) and prostate (C61) cancers, respectively; whereas there were only 3, 23, 18 incident cases of oesophagus (C15), stomach (C16) and brain cancers, respectively.

Supplementary Table S2 shows the univariate relationships between confounders and depressive symptoms amongst incident cancer cases. Participants who were female, unmarried, diagnosed with cardiovascular or chronic respiratory diseases, or had vocational or primary education or below, were more likely to have depressive symptoms at baseline than those who were male, married, diagnosed without cardiovascular or chronic respiratory diseases, or who had a university degree. However, compared with non-alcohol consumers, alcohol consumers were less likely to have depressive symptoms at baseline.

### 3.2 Association between follow-up years and depressive symptoms

Table 2 shows the results of our fully-adjusted model describing the association between follow-up years and depressive symptoms amongst incident cancer cases. After controlling for covariates, years of follow-up were positively associated with severity of depressive symptoms. For each year of observation, patients with cancer were 1.07 (95%CI: 1.05–1.10) times more likely to be in the highest tertile of CES-D scores (whether pre- or post-diagnosis). We also found significant quadratic and cubic effects of year, suggesting a non-linear effect of year on depressive symptoms.

**Table 2:** Fully-adjusted multilevel models for associations between years of follow-up (over the whole period of observation) and depressive symptom scores amongst incident cancer cases (*N* = 1056).

Fixed effects	ORs (95%CIs)	P-values
Year	1.07 (1.05–1.10)	<0.001
Year^2^*	1.0020 (1.0001–1.0039)	0.037
Year^3^*	0.9997 (0.9994–0.9999)	0.005
Gender		
Men (reference)		
Women	1.45 (1.12–1.89)	0.005
Age		
<50 years (reference)		
50–59 years	1.26 (0.78–2.05)	0.341
60–69 years	1.11 (0.69–1.78)	0.671
≥70 years	0.90 (0.40–2.04)	0.802
Marital status		
Married/Cohabiting (reference)		
Single/Divorced/Separated	1.51 (1.09–2.10)	0.013
Widowed	2.41 (1.66–3.49)	<0.001
Education		
University degree (reference)		
Secondary education	0.89 (0.64–1.24)	0.493
Vocational education	1.11 (0.80–1.55)	0.539
Primary education or below	1.93 (1.23–3.04)	0.004
Smoking		
Non-smokers (reference)		
Previous smokers	0.83 (0.64–1.09)	0.188
Current smokers (<1 cigarette per day)	0.62 (0.27–1.40)	0.246
Current smokers (≥1 cigarette per day)	0.96 (0.72–1.28)	0.777
Alcohol consumption		
Non-alcohol consumers (reference)		
<1 time per month	0.95 (0.64–1.42)	0.818
1–3 times per month	0.88 (0.58–1.33)	0.543
1–4 times per week	1.05 (0.70–1.58)	0.820
≥5 times per week	0.77 (0.49–1.22)	0.271
Physical activity (hours per week)	1.00 (0.99–1.01)	0.615
Fruit consumption (portions per day)	0.97 (0.94–1.00)	0.084
Vegetable consumption (portions per day)	0.99 (0.94–1.05)	0.856
Diabetes		
No (reference)		
Yes	1.27 (0.91–1.77)	0.167
Cardiovascular disease		
No (reference)		
Yes	2.00 (1.46–2.74)	<0.001
Chronic respiratory diseases		
No (reference)		
Yes	1.69 (1.25–2.29)	0.001
Body Mass Index		
Normal weight (BMI 18.5 to 24.9) (reference)		
Pre-obesity (BMI 25.0 to 29.9)	1.04 (0.78–1.40)	0.791
Obesity (BMI ≥ 30.0)	1.21 (0.88–1.67)	0.232
Thresholds	Log-odds	S.E.
Cut1	0.08 (−0.64–0.79)	0.36
Cut2	1.71 (0.99–1.91)	0.37
Random effects	Variance (95%CI)	S.E.
Individual level	1.55 (1.26–1.91)	0.16

OR: Odds Ratio; 95%CI: 95% Confidence Interval; BMI: Body Mass Index; S.E.: Standard Error.

⁎ Year^2^: quadratic term of year; Year^3^: cubic term of year.

**Fig. 2:**
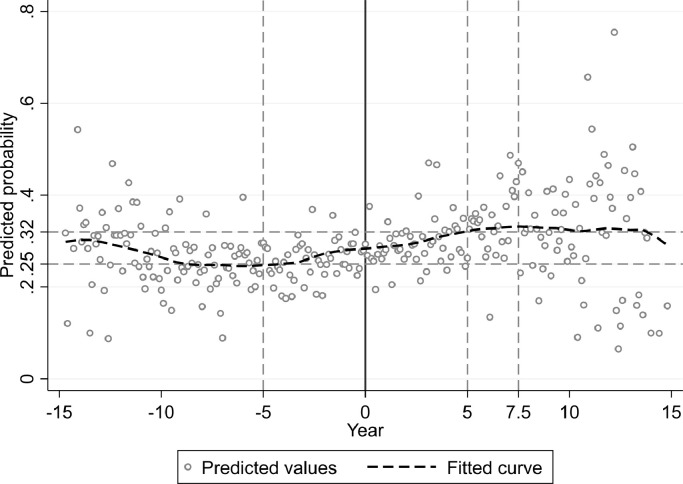
Predicted probabilities of being in the highest tertile of CES-D scores over time amongst incident cancer cases (*N* = 1065) CES-D: Centre for Epidemiological Studies-Depression.

Fig. 2 illustrates the predicted probability of being in the highest tertile of CES-D scores during follow-up amongst incident cancer cases, with year=0 as a proxy for cancer diagnosis. Overall, although the change in probability between two subsequent years was small, the cubic shape of the curve was statistically significant (Table 2, 0.9997, 95%CI: 0.9994–0.9999). The probability was around 25% at five years before hospitalisation, and increased to around 32% at five years after hospitalisation. Thereafter, the probability continued increasing at a slower rate until its highest point (around 33%) at 7.5 years after hospitalisation. The predicted values prior to 10 years pre-hospitalisation or after 10 years post-hospitalisation were dispersed, due to the small sample size for those followed-up for more than 10 years before or after hospitalisation (Supplementary Figure S3). The fitted curve prior to 10 years pre- or 10 years post-diagnosis could therefore be subject to biased interpretation.

### 3.3 Independent effect of cancer on depressive symptoms

Table 3 presents the results of multilevel models comparing incident cancer cases and matched cancer-free controls. Patients with cancer were at a significantly increased risk of high depressive symptom burden over time, whether in linear (1.07, 95%CI: 1.05–1.10) or non-linear models. In the cancer-free group, both linear and non-linear effects of follow-up year were non-significant. Supplementary Figure S4 compares the predicted probabilities of being in the highest tertile of CES-D scores after hospitalisation between cancer and cancer-free groups. For the cancer-free group, the probability did not change over time, but for the incident cancer group, it increased for around five years post- hospitalisation, and thereafter continued increasing but at a slower rate, to its highest point at 7.5 years. Participants with cancer had significantly greater probabilities of higher depressive symptoms than cancer-free controls from around three years to twelve years post- hospitalisation, suggesting an independent effect of cancer on depressive symptoms after hospitalisation.

**Table 3:** Associations between follow-uptime (years) and depressive symptoms in cancer (*N* = 1056) and cancer-free* (*N* = 1056) groups.

	Incident cancer cases (cancer group)		Matched individuals (cancer-free group)	
Fixed effects	ORs (95%CIs)	P-values	ORs (95%CIs)	P-values
Year	1.07 (1.05–1.10)	<0.001	1.02 (1.00–1.05)	0.056
Year^2^	1.0019 (1.0001–1.0038)	0.044	1.0012 (0.9995–1.0029)	0.167
Year^3^	0.9997 (0.9994–0.9999)	0.006	1.0000 (0.9997–1.0002)	0.428
Thresholds	Log-odds	S.E.	Log-odds	S.E.
Cut1	−0.51 (−0.65–−0.37)	0.07	−0.52 (−0.66–−0.37)	0.07
Cut2	1.11 (0.97–1.25)	0.07	1.10 (0.96–1.25)	0.07
Random effects	Variance (95%CI)	S.E.	Variance (95%CI)	S.E.
Individual level	1.87 (1.53–2.27)	0.19	2.24 (1.87–2.68)	0.20

OR: Odds Ratio; 95%CI: 95% Confidence Interval; S.E.: Standard Error.

⁎ Each cancer free control was allocated a non-exposure data, which was the date of first cancer hospitalisation of his/her matched cancer case.

### 3.4 Slope change of the probabilities of trajectory of depressive symptoms

The results of piecewise regression (Table 4) found that the slope change of the curve was statistically significant at five years before hospitalisation (0.12, 95%CI: 0.07–0.18), confirming that the probability of being in the highest tertile of CES-D scores started to increase at a greater rate at five years before hospitalisation. Although the probability continued to increase after year of hospitalisation (0.07, 95%CI: 0.01–0.13), this rate of change was not statistically significant (−0.05, 95%CI: −0.12–0.13).

**Table 4:** Changes of the probability of being in the highest tertile of CES-D scores at 6–15 and 1–4 years before hospitalisation s, and 1–15 years after hospitalisation.

Probabilities	Slope (change per year)	95%CIs	P-values
6–15 years before hospitalisation	−0.06	(−0.10–−0.02)	0.007
1–4 years before hospitalisation	0.11	(0.07–0.15)	<0.001
Change (between 6 and 15 years and 1–4 years before hospitalisation)	0.12	(0.07–0.18)	<0.001
1–15 years after hospitalisation	0.07	(0.01–0.13)	0.024
Change (between 1 and 4 years before hospitalisation and 1–15 years after hospitalisation)	−0.05	(−0.12–0.03)	0.250

CES-D: Centre for Epidemiological Studies-Depression; 95%CI: 95% Confidence Interval.

### 3.5 Sensitivity analyses

Compared with participants included in our analysis, excluded participants had lower education and consumed less alcohol, but were more likely to have CVD and high CES-D scores at baseline (Supplementary Table S3). Besides, we found similar results when using the z-scores of CES-D to test the relationship between follow-up years and depressive symptoms (Supplementary Table S4).

## 4 Discussion

### 4.1 Main findings

In this 16-year cohort study of 1056 incident cancer cases, we found a positive association between years of follow-up and depressive symptoms, covering the period both before and after the date of the first hospitalisation for cancer that we used as a proxy for cancer diagnosis. We found that the risk of depressive symptoms started increasing at five years before hospitalisation, and continued to increase until 7.5 years after hospitalisation. The most likely explanation is that our proxy for cancer diagnosis (the first hospitalisation for cancer) was much later than actual cancer diagnosis. However, even assuming a lag time of one year from diagnosis to the first hospitalisation, a four-year period of excess risk for depression prior to diagnosis is surprising, and highly suggestive of direct neuropsychiatric explanations. Using PSM, we confirmed that cancer was an independent predictor of depressive symptoms.

### 4.2 Results in the context of other studies

Other work has found the risk of mental disorders to increase prior to cancer diagnosis, including analysis of Swedish registers (Lu et al., 2016), in which risk of depression, anxiety, substance abuse, somatoform/conversion disorder, and stress reaction/adjustment disorder increased from 10 months before cancer diagnosis, peaked during the first week after diagnosis, and decreased rapidly thereafter, but remained elevated 10 years after diagnosis. Our findings also provide one explanation for the increased risk of suicide after a cancer diagnosis (Henson et al., 2018). Our finding of an excess risk of depressive disorders even prior to hospitalisation (used as a proxy for diagnosis), is consistent with previous work (Lu et al., 2016), prompting speculation about potential explanations. A pre-diagnostic phase of cancer-related systemic inflammation has been proposed (Mallet et al., 2017), leading to microglial dysfunction that is associated with psychiatric disorders including depression (Diakos et al., 2014; Krishnan and Nestler, 2008; Reus et al., 2015). It is possible that systemic inflammation contributes to the risk of depressive symptoms, effecting behavioural, affective and cognitive changes that are consistent with major depressive symptoms (Krishnan and Nestler, 2008; Messay et al., 2012).

Explanations involving systemic inflammatory effects are somewhat weakened by the similarities in risk of mental disorders before cancer diagnosis when comparing patients with localised or non-metastatic cancers and those with locally advanced or metastatic cancers (Lu et al., 2016). An alternative physiological explanation is that non-inflammatory cancer symptoms before diagnosis, such as anaemia, are associated with depression (Onder et al., 2005). A Swedish study estimated the risk of cancer in relation to haemoglobin concentration (a marker of anaemia) during the five years before cancer diagnosis, finding a long-duration of haemoglobin decline before cancer diagnosis: haemoglobin concentration had started declining three years before cancer diagnosis for malignancies including stomach cancer, multiple myeloma and lymphatic leukaemia (Edgren et al., 2010). It is also possible that depressive symptoms are the presenting complaint for specific cancers with neuropsychiatric effects (Benros et al., 2009), including pancreatic cancer, pituitary tumours, and non-small cell lung cancer (Makrilia et al., 2009; Pitman et al., 2018), although these are in the minority.

Biopsychosocial explanations are also possible, including the effect of rumination about pre-diagnostic cancer symptoms and the psychological stress of undergoing clinical evaluation for a suspected malignancy (Lu et al., 2017). Psychological stress relating to a cancer diagnosis can itself can have physiological effects (such as sustained stress causing activation of the hypothalamo-pituitary-adrenal axis), giving rise to psychological symptoms that can reach diagnostic thresholds for depression (Pitman et al., 2018). Psychological stress following cancer diagnosis, including the stress of treatment, might also activate inflammatory pathways, with a bidirectional association with low mood (Messay et al., 2012).

### 4.3 Strengths and limitations

We used a population-based representative sample to investigate an important yet under-researched clinical question in a geographical region with a high prevalence of alcohol-related risk factors for cancer (World Health Organization, 2018). Using routine clinical data, we examined time trends over a period of up to 15 years prior to the first hospitalisation for cancer, and 15 years after the first hospitalisation, including a validated measure of depressive symptoms (Kohout et al., 1993; Radloff, 1977). We used advanced statistical methods, including multilevel modelling, to predict the trajectory of the probabilities of depressive symptoms before and after the first hospitalisation for cancer, piecewise regression to quantify the slope changes in this trajectory, and propensity score matched controls to explore whether cancer was an independent risk factor for depression.

This study has several limitations. Firstly, we excluded 181 incident cancer cases due to missing data for depressive symptoms and confounders. Given the differences between included and excluded cases, it is possible that our analysis underestimated the effects of education, alcohol consumption and CVD on depressive symptoms.

Secondly, due to a lack of dates of cancer diagnosis in outpatient clinics, we ascertained cancer diagnosis using the first hospitalisation for cancer, thereby defining the date of the first cancer hospitalisation as a proxy for cancer diagnostic date may have been later than the date of cancer diagnosis at outpatient clinics, particularly for cancers of the blood and skin. However, even assuming a lag time of one year from outpatient diagnosis to the first hospitalisation, a four-year period of excess risk for depression prior to cancer diagnosis from our analysis, is highly suggestive of direct neuropsychiatric explanations.

Thirdly, stratified analysis by cancer types was not conducted due to the small sample size for each type of cancer, although this variable was available (Supplementary Figure S2). In the Czech Republic, first hospitalisation for cancer can occur at any of I-III clinical stages of cancer (National Cancer Control Programme, 2011). However, we lacked a measure of cancer stage at first hospitalisation, which is likely to influence psychological health and suicide risk (Bjorkenstam et al., 2005). Information on antidepressant prescriptions was unknown. Antidepressants may influence cancer risk by affecting the immune system, representing as a potential confounder of the cancer – depression relationship (Steingart and Cotterchio, 1995). We also lacked a measure of the proportion of admissions involving emergency presentations (Herbert et al., 2019), so could not include these in our interaction tests. Variations in cancer types, stages of malignancy, antidepressant use and emergency presentations could explain the individual-level residuals in random effects in the fully-adjusted model in Table 2. Future stratified analyses by cancer types and stages of malignancy based on cohort studies with larger sample sizes are needed.

Fourthly, our outcome was a self-rated measure of depressive symptoms rather than a clinical diagnosis of depression, creating the potential for reporting bias. However, the CES-D scale is capable of distinguishing the severity of depression and providing valid information on which to base decisions about psychiatric treatment (Kohout et al., 1993; Radloff, 1977). We harmonised sum CES-D scores using tertiles, making it more difficult to compare our results with other samples, as tertiles were dependant on the dataset used for the current analysis (Bennette and Vickers, 2012). However, we found similar results for the main relationship when using z-scores of CES-D (Supplementary Table S5), suggesting that different harmonisation strategies would not bring bias for the true relationship. Our findings are also consistent with previous evidence (Lu et al., 2016).

Lastly, our univariate analysis found that alcohol consumers were less likely to have depressive symptoms than non-alcohol consumers at baseline. Previous studies have suggested a U-shaped relationship between frequency of alcohol consumption and mortality amongst US middle-aged and older adults (Thun et al., 1997) and British men (Marmot et al., 1981) as well as in relation to psychological distress amongst young British adults (Power et al., 1998). One explanation for this pattern is that the low consumption group includes former heavy drinkers, who stopped drinking due to worsening health or chronic medical conditions, and who may also be at higher risk of mental health problems (Boden and Fergusson, 2011). It is possible that in our study non-alcohol consumers included abstainers who are more likely to have severe depressive symptoms associated with previous misuse than the current alcohol consumers. However, 15% of patients with cancer in this study consumed alcohol more than 5 times per week, suggesting that this group were likely to have an alcohol misuse disorder. Our categorical variable describing alcohol use provided little detail on quantities consumed, and relied on the assumption of each drinking session being equivalent. Without greater granularity in this variable, and also lacking a variable describing presence or absence of alcohol misuse disorder, we cannot infer the reasons for this surprising association.

### 4.4 Clinical implications

To our knowledge, this is the first study examining risk of depressive symptoms before and after the first hospitalisation for cancer in a Central and Eastern European country. Our findings suggest that depressive symptoms emerge five years before the first cancer hospitalisation, for which no biological or biopsychosocial explanations are confirmed. Our findings highlight the need for early psychological assessment of patients diagnosed with cancer to assess their support needs. Identifying and treating depression has the potential to improve quality of life, adherence to treatment, cancer survival, and treatment costs (Pitman et al., 2018). Evidence-based models of care include the provision of integrated psychological support service (specialist counsellors and clinical psychologists embedded in cancer services), providing collaborative screening and treatment (Sharpe et al., 2014). However, even in well-resourced countries these models of care tend not to be available outside major urban centres (Pitman et al., 2018). Integrating mental healthcare into physical healthcare settings is also in keeping with the WHO's Mental Health Action Plan 2013–2020, especially in low and middle-income countries (Stein et al., 2019).

## 5 Conclusions

In conclusion, we found that cancer was an independent predictor of high depressive symptom burden in a representative sample of adults in the Czech Republic. This risk was apparent from five years prior to the first hospitalisation for cancer, and continued for up to 7.5 years after the first hospitalisation.

## Funding

Wentian Lu and Martin Bobak are supported by the European Union's 10.13039/501100007601Horizon 2020 Research and Innovation Programme (grant 635316) and the European Commission 10.13039/501100007601Horizon 2020 (grant 667661). Alexandra Pitman is supported by the 10.13039/501100012618National Institute of Health Research (10.13039/501100000272NIHR) University College London Hospitals (UCLH) Biomedical Research Centre (BRC). HAPIEE has been funded by the 10.13039/100004440Wellcome Trust (grants 064947 and 081081), the 10.13039/100000001US National Institute on aging (grant R01 AG23522-01), and the 10.13039/100000870MacArthur Foundation ‘Initiative on Social Upheaval and Health’ (grant 712058). None of the funding bodies had any role in study design, data collection, data analysis, data interpretation, or writing of the report.

## Contributors

Wentian Lu and Martin Bobak designed the study. Wentian Lu performed the statistical analysis and wrote the first draft of the article. Martin Bobak, Alexandra Pitman and Hynek Pikhart assisted Wentian Lu with refining the analysis and interpreting results. Martin Bobak, Alexandra Pitman, Hynek Pikhart, Anne Peasey and Ruzena Kubinova all assisted with editing the article.

## Declaration of Competing Interest

None.

## Appendix Supplementary materials

Image, application 1
