# Supplementary material for: Risk of depressive symptoms before and after the first hospitalisation for cancer: Evidence from a 16-year cohort study in the Czech Republic
Source: J Affect Disord. 2020 Nov 1;276:76–83. doi: 10.1016/j.jad.2020.06.070 (PMC7456789; doi:10.1016/j.jad.2020.06.070)
Supplement: Supplementary file 1 [file mmc1.docx]

# **Supplementary documents**

***Figure S1 Numbers of incident cancer cases over period of interest (2003-2017, N=1056)***

***
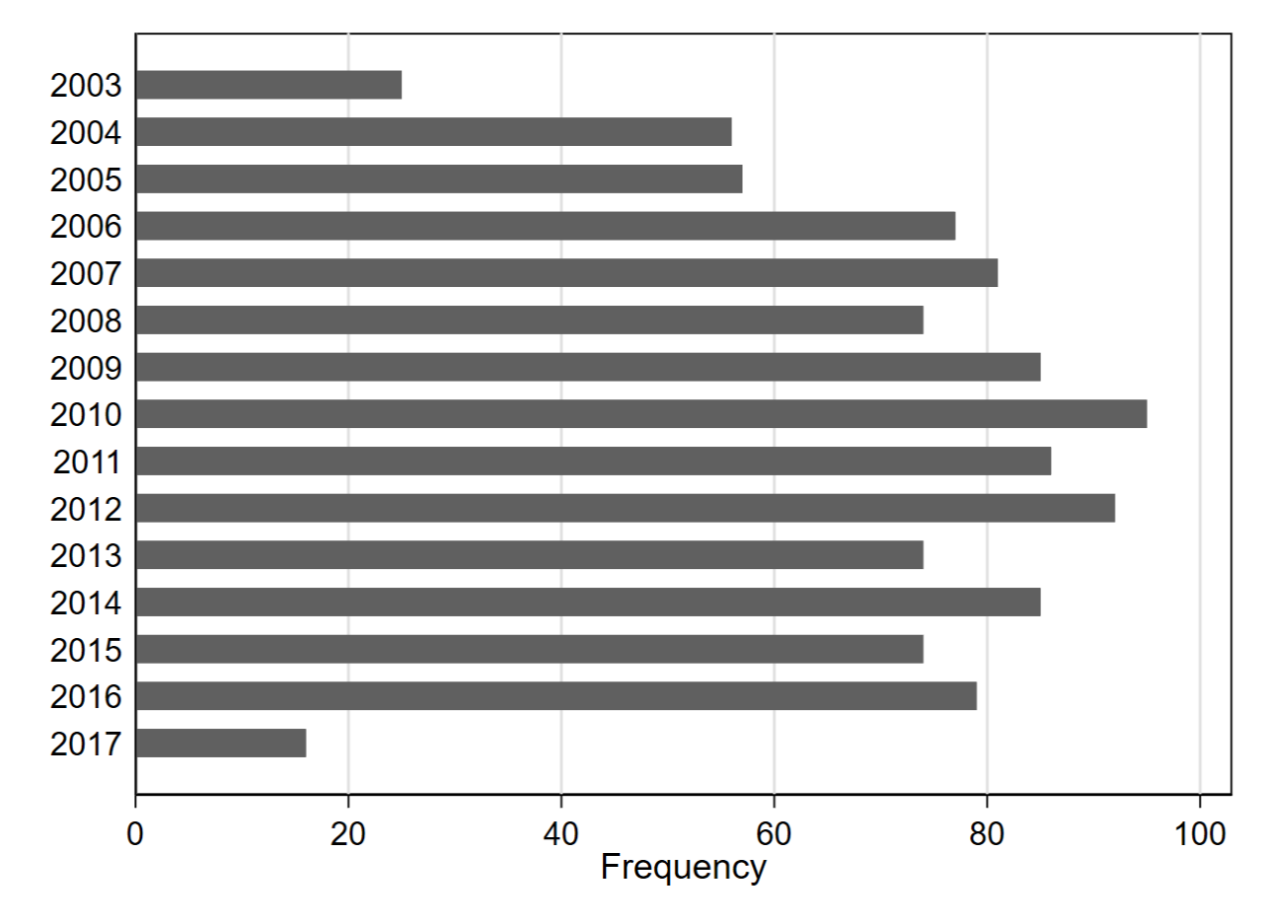
***

***Figure S2 Numbers of each type of cancer (N=1056)***

**
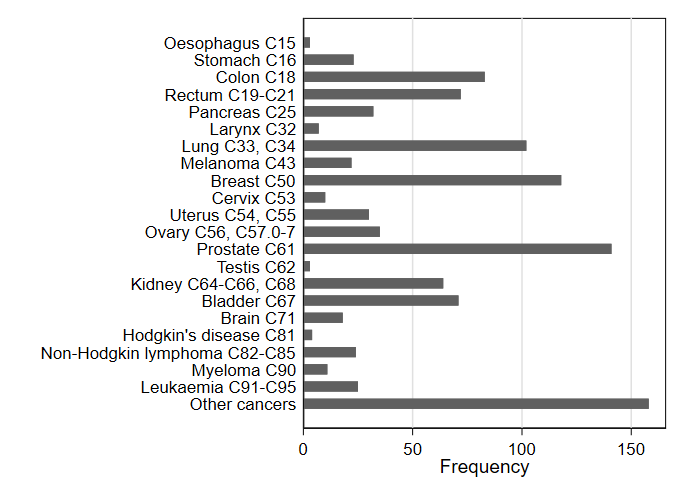
**

***Figure S3 Distribution of follow-up years before and after the first hospitalisation for cancer among incident cancer cases (N=1056)***

***
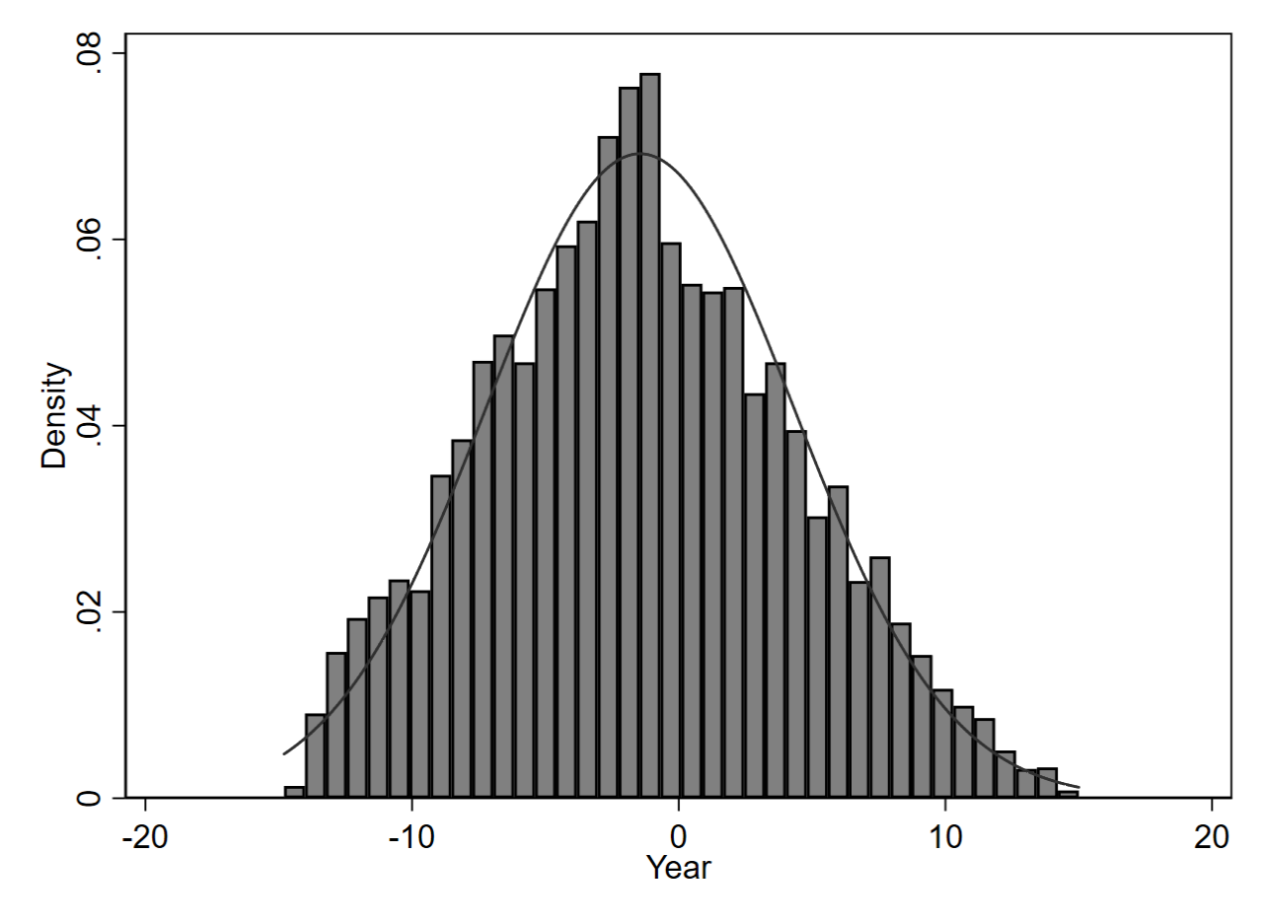
***

***Figure S4 Predicted probabilities of being in the highest tertile of CES-D scores after the first hospitalisation for cancer among patients with cancer (N=1056) and matched cancer-free (N=1056) participants***

***
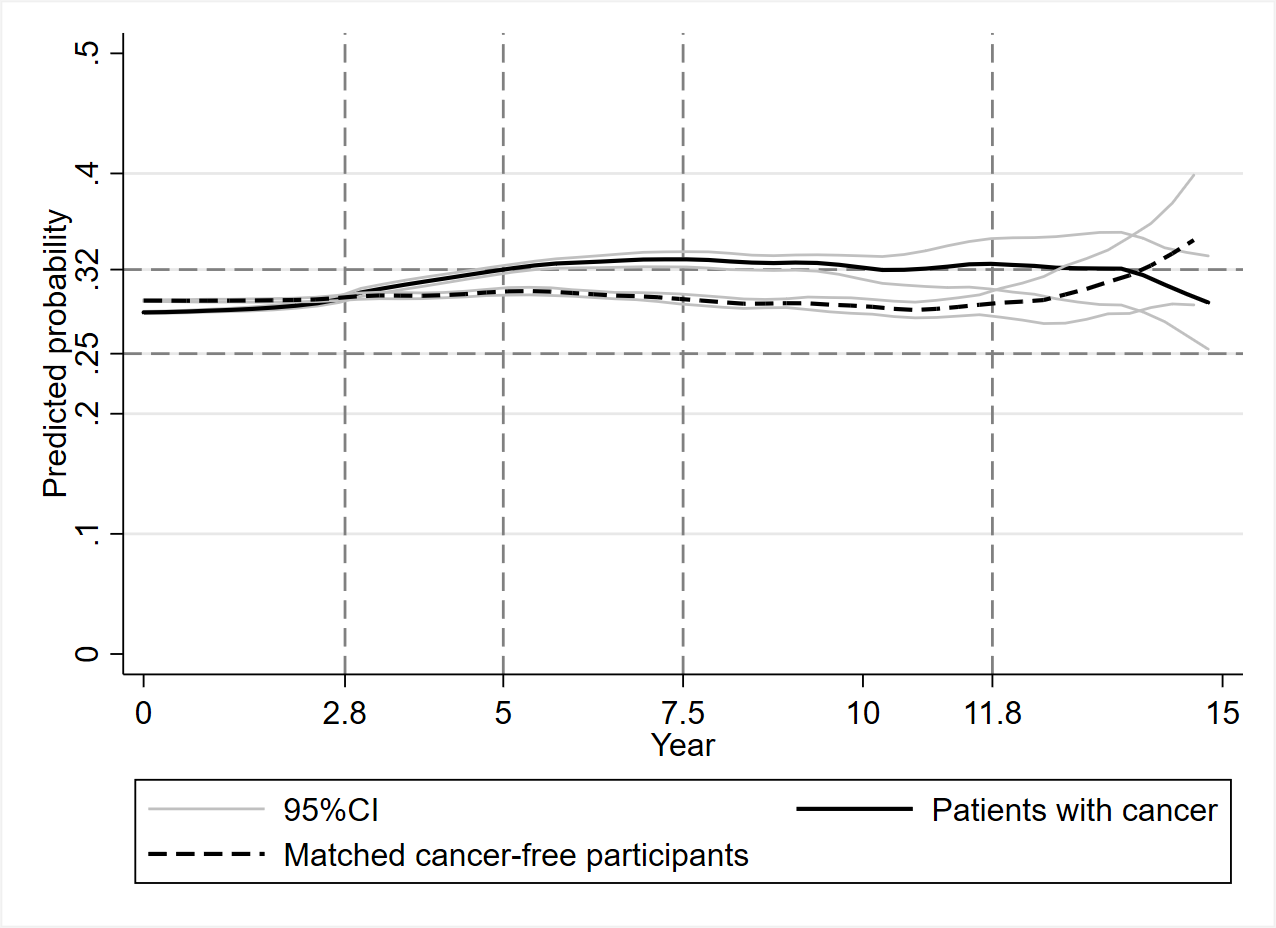
***

*CES-D: Centre for Epidemiological Studies-Depression*

***Table S1 Comparing baseline characteristics between incident cancer cases (N=1056) and matched cancer-free controls (N=1056)***

|  | Incident cancer cases | Matched controls | P-values |
| --- | --- | --- | --- |
| CES-D scores (%) |  |  |  |
| Lowest tertile (reference) | 37.59 | 39.68 | 0.616 |
| Middle tertile | 34.85 | 33.62 |  |
| Highest tertile | 27.56 | 26.70 |  |
| Gender (%) |  |  |  |
| Men (reference) | 55.11 | 55.21 | 0.965 |
| Women | 44.89 | 44.79 |  |
| Age (%) |  |  |  |
| <50 years (reference) | 8.62 | 6.16 | 0.154 |
| 50-59 years | 31.06 | 32.10 |  |
| 60-69 years | 57.20 | 59.09 |  |
| ≥70 years | 3.13 | 2.65 |  |
| Marital status (%) |  |  |  |
| Married/Cohabiting (reference) | 75.47 | 74.91 | 0.948 |
| Single/Divorced/Separated | 14.02 | 14.20 |  |
| Widowed | 10.51 | 10.89 |  |
| Education (%) |  |  |  |
| University degree (reference) | 14.30 | 13.83 | 0.460 |
| Secondary education | 33.71 | 35.04 |  |
| Vocational education | 36.84 | 38.26 |  |
| Primary education or below | 15.15 | 12.88 |  |
| Smoking (%) |  |  |  |
| Non-smokers (reference) | 37.78 | 37.88 | 0.249 |
| Previous smokers | 30.11 | 32.10 |  |
| Current smokers (<1 cigarette per day) | 3.03 | 1.80 |  |
| Current smokers (≥1 cigarette per day) | 29.07 | 28.22 |  |
| Alcohol consumption (%) |  |  |  |
| Non-alcohol consumers (reference) | 13.45 | 12.31 | 0.943 |
| <1 time per month | 24.34 | 25.28 |  |
| 1-3 times per month | 20.93 | 20.64 |  |
| 1-4 times per week | 26.04 | 26.33 |  |
| ≥5 times per week | 15.25 | 15.44 |  |
| Physical activity (hours per week) |  |  |  |
| Mean (S.D.) | 13.88 (12.86) | 13.49 (12.50) | 0.480 |
| Fruit consumption (portions per day) |  |  |  |
| Mean (S.D.) | 3.45 (3.60) | 3.48 (3.52) | 0.855 |
| Vegetable consumption (portions per day) |  |  |  |
| Mean (S.D.) | 3.10 (2.75) | 3.13 (2.25) | 0.753 |
| Diabetes (%) |  |  |  |
| No (reference) | 86.08 | 86.84 | 0.611 |
| Yes | 13.92 | 13.16 |  |
| Cardiovascular disease (%) |  |  |  |
| No (reference) | 84.56 | 83.62 | 0.552 |
| Yes | 15.44 | 16.38 |  |
| Chronic respiratory diseases (%) |  |  |  |
| No (reference) | 83.33 | 83.05 | 0.861 |
| Yes | 16.67 | 16.95 |  |
| Body Mass Index (%) |  |  |  |
| Normal weight (BMI 18.5 to 24.9) (reference) | 19.22 | 20.83 | 0.636 |
| Pre-obesity (BMI 25.0 to 29.9) | 45.27 | 44.79 |  |
| Obesity (BMI ≥ 30.0) | 35.51 | 34.38 |  |

*CES-D: Centre for Epidemiological Studies-Depression; S.D.: Standard Deviation; BMI: Body Mass Index*

***Table S2 Univariate associations between confounders and depressive symptoms among incident cancer cases (N=1056)***

| Risk factors | Total | ORs (95%CIs) | P-values |
| --- | --- | --- | --- |
| Gender (%) |  |  |  |
| Men (reference) | 55.21 |  |  |
| Women | 44.79 | 1.50 (1.20 – 1.88) | <0.001 |
| Age (%) |  |  |  |
| <50 years (reference) | 6.16 |  |  |
| 50-59 years | 32.10 | 0.88 (0.53 – 1.45) | 0.612 |
| 60-69 years | 59.09 | 0.76 (0.47 – 1.23) | 0.270 |
| ≥70 years | 2.65 | 0.51 (0.22 – 1.18) | 0.118 |
| Marital status (%) |  |  |  |
| Married/Cohabiting (reference) | 74.91 |  |  |
| Single/Divorced/Separated | 14.20 | 1.73 (1.25 – 2.38) | 0.001 |
| Widowed | 10.89 | 2.42 (1.67 – 3.51) | <0.001 |
| Education (%) |  |  |  |
| University degree (reference) | 13.83 |  |  |
| Secondary education | 35.04 | 1.32 (0.92 – 1.91) | 0.131 |
| Vocational education | 38.26 | 1.48 (1.03 – 2.12) | 0.032 |
| Primary education or below | 12.88 | 2.46 (1.58 – 3.83) | <0.001 |
| Smoking (%) |  |  |  |
| Non-smokers (reference) | 37.88 |  |  |
| Previous smokers | 32.10 | 0.88 (0.68 – 1.15) | 0.361 |
| Current smokers (<1 cigarette per day) | 1.80 | 0.83 (0.37 – 1.86) | 0.645 |
| Current smokers (≥1 cigarette per day) | 28.22 | 0.97 (0.74 – 1.29) | 0.849 |
| Alcohol consumption (%) |  |  |  |
| Non-alcohol consumers (reference) | 12.31 |  |  |
| <1 time per month | 25.28 | 0.65 (0.44 – 0.96) | 0.031 |
| 1-3 times per month | 20.64 | 0.69 (0.46 – 1.03) | 0.067 |
| 1-4 times per week | 26.33 | 0.64 (0.44 – 0.94) | 0.023 |
| ≥5 times per week | 15.44 | 0.47 (0.31 – 0.72) | <0.001 |
| Physical activity (hours per week) |  |  |  |
| Mean (S.D.) | 13.49 (12.50) | 0.99 (0.98 – 1.00) | 0.158 |
| Fruit consumption (portions per day) |  |  |  |
| Mean (S.D.) | 3.48 (3.52) | 0.99 (0.96 – 1.02) | 0.426 |
| Vegetable consumption (portions per day) |  |  |  |
| Mean (S.D.) | 3.13 (2.25) | 0.99 (0.95 – 1.04) | 0.793 |
| Diabetes (%) |  |  |  |
| No (reference) | 86.84 |  |  |
| Yes | 13.16 | 1.14 (0.82 – 1.59) | 0.423 |
| Cardiovascular disease (%) |  |  |  |
| No (reference) | 83.62 |  |  |
| Yes | 16.38 | 1.32 (0.98 – 1.78) | 0.071 |
| Chronic respiratory diseases (%) |  |  |  |
| No (reference) | 83.05 |  |  |
| Yes | 16.95 | 1.94 (1.44 – 2.61) | <0.001 |
| Body Mass Index (%) |  |  |  |
| Normal weight (BMI 18.5 to 24.9) (reference) | 20.83 |  |  |
| Pre-obesity (BMI 25.0 to 29.9) | 44.79 | 0.87 (0.64 – 1.17) | 0.350 |
| Obesity (BMI ≥ 30.0) | 34.38 | 0.91 (0.66 – 1.24) | 0.551 |

*OR: Odds Ratio; 95%CI: 95% Confidence Interval; S.D.: Standard Deviation; BMI: Body Mass Index*

***Table S3 Comparing baseline characteristics between analysed (N=1056) and excluded incident cancer cases (N=181)***

|  | Analysed cases | Excluded cases | P-values |
| --- | --- | --- | --- |
| CES-D scores (%) |  |  |  |
| Lowest tertile (reference) | 39.68 | 27.55 | 0.017 |
| Middle tertile | 33.62 | 33.67 |  |
| Highest tertile | 26.7 | 38.78 |  |
| Gender (%) |  |  |  |
| Men (reference) | 55.21 | 56.35 | 0.775 |
| Women | 44.79 | 43.65 |  |
| Age |  |  |  |
| <50 years (reference) | 6.16 | 5.52 | 0.983 |
| 50-59 years | 32.10 | 33.15 |  |
| 60-69 years | 59.09 | 58.56 |  |
| ≥70 years | 2.65 | 2.76 |  |
| Marital status (%) |  |  |  |
| Married/Cohabiting (reference) | 74.91 | 72.16 | 0.726 |
| Single/Divorced/Separated | 14.20 | 15.34 |  |
| Widowed | 10.89 | 12.50 |  |
| Education (%) |  |  |  |
| University degree (reference) | 13.83 | 9.36 | 0.031 |
| Secondary education | 35.04 | 27.49 |  |
| Vocational education | 38.26 | 46.20 |  |
| Primary education or below | 12.88 | 16.96 |  |
| Smoking (%) |  |  |  |
| Non-smokers (reference) | 37.88 | 36.14 | 0.754 |
| Previous smokers | 32.10 | 35.54 |  |
| Current smokers (<1 cigarette per day) | 1.80 | 2.41 |  |
| Current smokers (≥1 cigarette per day) | 28.22 | 25.90 |  |
| Alcohol consumption (%) |  |  |  |
| Non-alcohol consumers (reference) | 12.31 | 22.14 | 0.008 |
| <1 time per month | 25.28 | 29.77 |  |
| 1-3 times per month | 20.64 | 17.56 |  |
| 1-4 times per week | 26.33 | 20.61 |  |
| ≥5 times per week | 15.44 | 9.92 |  |
| Physical activity (hours per week) |  |  |  |
| Mean (S.D.) | 13.49 (12.50) | 11.77 (11.60) | 0.127 |
| Fruit consumption (portions per day) |  |  |  |
| Mean (S.D.) | 3.48 (3.52) | 3.95 (6.49) | 0.157 |
| Vegetable consumption (portions per day) |  |  |  |
| Mean (S.D.) | 3.13 (2.25) | 2.96 (2.57) | 0.356 |
| Diabetes (%) |  |  |  |
| No (reference) | 86.84 | 82.78 | 0.144 |
| Yes | 13.16 | 17.22 |  |
| Cardiovascular disease (%) |  |  |  |
| No (reference) | 83.62 | 72.41 | 0.001 |
| Yes | 16.38 | 27.59 |  |
| Chronic respiratory diseases (%) |  |  |  |
| No (reference) | 83.05 | 76.74 | 0.076 |
| Yes | 16.95 | 23.26 |  |
| Body Mass Index (%) |  |  |  |
| Normal weight (BMI 18.5 to 24.9) (reference) | 20.83 | 20.00 | 0.754 |
| Pre-obesity (BMI 25.0 to 29.9) | 44.79 | 47.78 |  |
| Obesity (BMI ≥ 30.0) | 34.38 | 32.22 |  |

*CES-D: Centre for Epidemiological Studies-Depression; S.D.: Standard Deviation; BMI: Body Mass Index*

***Table S4 Results of a fully-adjusted multilevel model for the association between follow-up years and depressive symptoms (z-scores) among incident cancer cases (N=1056)***

|  | b (95%CIs) | P-values |
| --- | --- | --- |
| Fixed effects |  |  |
| Year | 0.03 (0.02 – 0.04) | <0.001 |
| Year^2^ | 0.00 (0.00 – 0.00) | 0.568 |
| Year^3^ | 0.00 (0.00 – 0.00) | 0.002 |
| Gender |  |  |
| Men (reference) |  |  |
| Women | 0.17 (0.06 – 0.27) | 0.002 |
| Age |  |  |
| <50 years (reference) |  |  |
| 50-59 years | 0.14 (-0.06 – 0.33) | 0.164 |
| 60-69 years | 0.07 (-0.12 – 0.25) | 0.497 |
| ≥70 years | -0.08 (-0.41 – 0.25) | 0.628 |
| Marital status |  |  |
| Married/Cohabiting (reference) |  |  |
| Single/Divorced/Separated | 0.16 (0.03 – 0.29) | 0.018 |
| Widowed | 0.35 (0.21 – 0.50) | <0.001 |
| Education |  |  |
| University degree (reference) |  |  |
| Secondary education | -0.08 (-0.22 – 0.05) | 0.218 |
| Vocational education | 0.00 (-0.13 – 0.14) | 0.962 |
| Primary education or below | 0.30 (0.12 – 0.48) | 0.001 |
| Smoking |  |  |
| Non-smokers (reference) |  |  |
| Previous smokers | -0.06 (-0.17 – 0.05) | 0.310 |
| Current smokers (<1 cigarette per day) | -0.19 (-0.52 – 0.14) | 0.270 |
| Current smokers (≥1 cigarette per day) | -0.02 (-0.13 – 0.10) | 0.798 |
| Alcohol consumption |  |  |
| Non-alcohol consumers (reference) |  |  |
| <1 time per month | -0.14 (-0.30 – 0.02) | 0.090 |
| 1-3 times per month | -0.15 (-0.32 – 0.01) | 0.070 |
| 1-4 times per week | -0.08 (-0.24 – 0.09) | 0.360 |
| ≥5 times per week | -0.16 (-0.35 – 0.02) | 0.081 |
| Physical activity (hours per week) | 0.00 (0.00 – 0.01) | 0.374 |
| Fruit consumption (portions per day) | -0.01 (-0.03 – 0.00) | 0.087 |
| Vegetable consumption (portions per day) | 0.00 (-0.02 – 0.02) | 0.882 |
| Diagnosed diabetes |  |  |
| No (reference) |  |  |
| Yes | 0.12 (-0.02 – 0.25) | 0.089 |
| Diagnosed cardiovascular disease |  |  |
| No (reference) |  |  |
| Yes | 0.22 (0.09 – 0.34) | 0.001 |
| Diagnosed chronic respiratory diseases |  |  |
| No (reference) |  |  |
| Yes | 0.25 (0.13 – 0.37) | <0.001 |
| Body Mass Index |  |  |
| BMI: Normal weight (reference) |  |  |
| BMI: Pre-obesity | -0.01 (-0.13 – 0.11) | 0.870 |
| BMI: Obesity | 0.06 (-0.07 – 0.19) | 0.340 |
| Intercept | -0.12 (-0.41 – 0.16) | 0.397 |
| Random effects | **Variance (95%CI)** | **S.E.** |
| Level 1: residual | 0.76 (0.74 – 0.78) | 0.01 |
| Level 2: intercept | 0.54 (0.50 – 0.58) | 0.02 |

*95%CI: 95% Confidence Interval; S.E.: Standard Error; BMI: Body Mass Index*
